# A Comparison of Endoscopic Closure and Laparoscopic Repair for Gastric Wall Defection

**DOI:** 10.1155/2022/9963126

**Published:** 2022-05-25

**Authors:** Qiao Qiao, Huiming Tu, Bojian Fei, Kebin Xu, Fan Yang, Jie Li, Qizhong Gao

**Affiliations:** ^1^Department of Gastroenterology, Affiliated Hospital of Jiangnan University, Wuxi, Jiangsu 214062, China; ^2^Department of Laparoscopic Surgery, Affiliated Hospital of Jiangnan University, Wuxi, Jiangsu 214062, China

## Abstract

**Objective:**

To compare the effectiveness and safety of endoscopic closure and laparoscopic repair for gastric wall defection.

**Method:**

The clinical data of 120 patients with submucosal tumours enrolled at our hospital between January 2014 and December 2019 were retrospectively analysed. Patients were divided into two groups according to the surgery they underwent: an endoscopic closure group (*n* = 60) and a laparoscopic repair group (*n* = 60). The clinical characteristics, perioperative complications, and postoperative follow-up results of the two groups were analysed.

**Results:**

The surgery time in the endoscopic closure group was 56.20 ± 11.25 minutes, which was significantly lower compared with that in the laparoscopic repair group (159.35 ± 23.18 minutes; *P* < 0.001). In addition, the postoperative stay in the endoscopic closure group was shorter than that in the laparoscopic repair group, and the intraoperative bleeding volume and incidence of enteral nutrition initiation after surgery were significantly lower. Medical expenses were also significantly lower in the endoscopic closure group than in the laparoscopic repair group (*P* < 0.001). Only one patient developed a postoperative fever in the endoscopic closure group; three patients developed a postoperative fever and one patient had postoperative bleeding in the laparoscopic repair group. However, there were no statistical differences between the two groups regarding the incidence of R0 resection, postoperative fever, postoperative bleeding, and closure failure (all *P* > 0.05). There were no local recurrences, distant metastases, or deaths in either of the groups during the two-year follow-up period.

**Conclusion:**

Non-laparoscopic-assisted surgery may be quicker, safer, and more effective for gastric wall defection.

## 1. Introduction

Due to the deep location of submucosal tumours that originate from the muscularis propria of the stomach, simple endoscopic treatment proves risky and difficult, the treatment effect is poor, and it is prone to various perforation complications [1, 2]. Based on the aim of removing the tumour completely, another difficulty must be considered for endoscopic resection; a full-layer resection can cause perforation, which will require laparoscopic repair in the early stage of endoscopic resection. In 2008, Zhou et al. [2, 3] first applied the endoscopic submucosal dissection (ESD) technique to the treatment of muscularis propria gastrointestinal stromal tumours (GISTs) and labelled it “endoscopic submucosal excavation” (ESE). In 2009 [4], researchers reported on endoscopic full-thickness resection (EFR). Once perforation occurs, endoscopic closure is performed quickly using titanium clips and nylon rope. In this procedure, perforation is different from complication perforation; that is, it is a process and step that is part of an endoscopic treatment known as “therapeutic perforation” [5–7]. The endoscopic resection of the gastric muscularis propria comprises three steps: ESE/EFR, therapeutic perforation, and endoscopic perforation closure. Although endoscopic perforation closure is completed [8–10], the closed wall is missing.

To date, a comparison of endoscopic closure and laparoscopic repair for gastric wall defection has not been presented. This study investigated and compared the effectiveness and safety of these procedures.

## 2. Materials and Methods

### 2.1. Subjects

A total of 120 patients who had submucosal tumours originating in the muscularis propria of the stomach, who sustained perforation during endoscopic resection, and who were enrolled at our hospital between January 2014 and December 2019 were included in this study. The patients were divided into two groups based on the type of procedure they underwent: an endoscopic closure group (*n* = 60) and a laparoscopic repair group (*n* = 60). The clinical characteristics, perioperative complications, and postoperative follow-up results of the two groups were analysed.

This study was conducted in accordance with the Declaration of Helsinki and approved by the ethics committee of our hospital. All participants provided signed informed consent for inclusion in the research.

### 2.2. Inclusion and Exclusion Criteria

The inclusion criteria were as follows: (1) cases of submucosal tumours (SMTs) originating from the gastric muscularis propria detected by endoscopic ultrasonography (EUS); (2) postoperative pathology included GISTs posing a very low or low risk; a positive immunohistochemistry result for CDll7 and DOG-1 was obtained; the GISTs were classified according to their clinical risk of malignancy (very low, low, moderate, and high [11]); and (3) perforation occurred during endoscopic submucosal tumour resection, causing gastric wall defection.

The exclusion criteria were as follows: (1) patients who had an advanced malignant tumour; (2) patients whose data were incomplete; and (3) the postoperative pathologic diagnosis being moderate or high risk.

### 2.3. Instruments

The therapeutic endoscopic procedure used a water injection endoscope (GIF-H260, Olympus, Japan), a double-cavity therapeutic gastroscope, a double-curved gastroscope, a double-lumen gastroscope, an OLYMPUS GIF-XQ 260 electronic gastroscope, an Olympus integrated nylon rope, a Leo hook nylon rope, disposable closed titanium clips (HX-600-135, Olympus, Japan), a titanium clip release device (HX-1 10ur, Olympus, Japan), and a Nanjing minimally invasive opening and closing harmony clip.

The ESD-related instruments included argon (ICC-200; ERBE, Germany), a dual knife, a transparent cap (Dmur201-11802, Olympus, Japan), thermal biopsy forceps (FD-410LR, Olympus, Japan), an injection needle (NM-4L-1, Olympus, Japan), and a carbon dioxide (CO_2_) injection pump (UCR, Olympus, Japan). The laparoscopic surgical instruments included a needle holder for the endoscope and 3.0 absorbable sutures.

### 2.4. Surgical Methods

#### 2.4.1. Preoperative Preparation

All patients fasted for eight hours before undergoing surgery. Prophylactic treatment with proton pump inhibitors and antibiotics was initiated two hours before the operation. A gastric tube was placed for continuous negative pressure suction to reduce the flow of gastric acid and content into the abdominal cavity. The umbilical hole was cleaned to prevent postoperative incision infection. Intraoperative therapeutic perforation was performed either by laparoscopic or endoscopic closure. All surgeries were performed by an endoscopist with more than 20 years of experience, including 2 assistant physicians and 1 anesthesiologist.

#### 2.4.2. Submucosal Tumour Resection

The operating room was arranged as a 10000-level clean double endoscope-combined operating room at a temperature of 22°C–25°C and with 40%–60% humidity. Endoscopy, combined with laparoscopic surgery, was performed under general anaesthesia delivered via intubation, with the patient positioned on their left side of the supine head. Before conducting the procedure, the gastric cavity was fully rinsed and gastric fluid was absorbed. During the procedure, the patient's vital signs and CO_2_ partial pressure were monitored. Pneumoperitoneum pressure was monitored while performing the therapeutic perforation. If an abdominal wall bulge and a drum sound were identified, a 20 ml injection needle was used to immediately reduce the abdominal pressure. The puncture point was located below the right costal margin and placed through the abdominal cavity until the perforation was completely closed. It was confirmed that no air had been discharged from the exhaust needle, which was removed when the pneumoperitoneum improved. Treatment to reduce the risk of postoperative peritonitis was then administered. Continuing on, ESE and EFR gastric wall tumour resection was conducted.

ESE tumour exhumation takes a long time and requires careful peeling off along the surface of the tumour; the perforation is often small and can easily be closed using a titanium clip. Perforation of the EFR gastric wall tumour resection, however, is large and obvious, and pneumoperitoneum can occur rapidly; it is thus necessary to monitor the abdominal exhaust of the perforation.

#### 2.4.3. Endoscopic Closure

**Figure 1:**
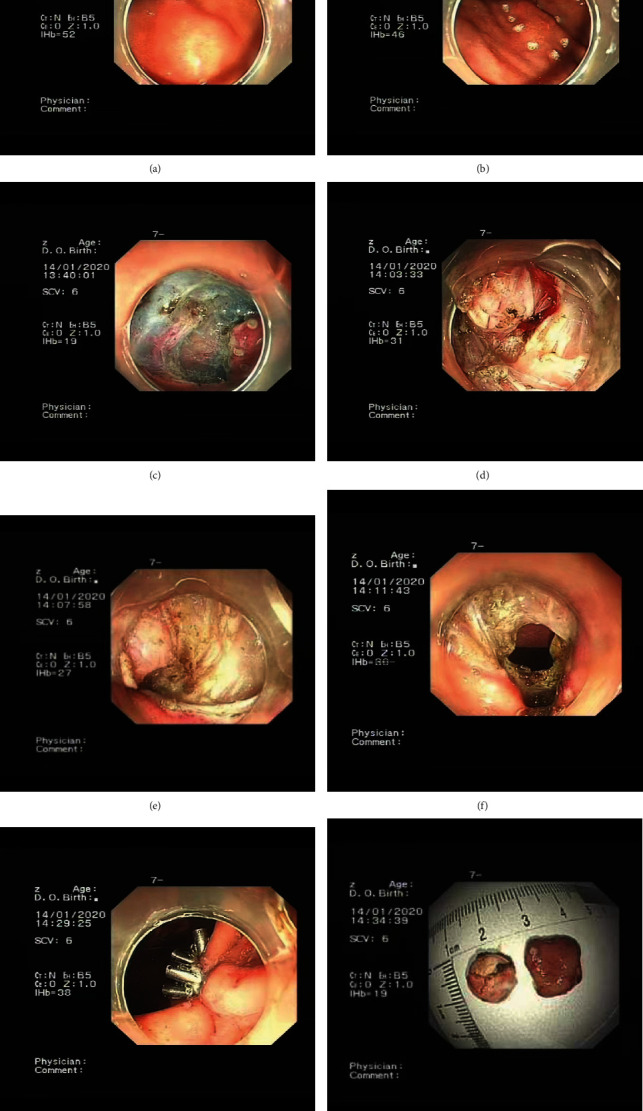
Endoscopic full-thickness resection and endoscopic closure procedure. (a) Endoscopic findings: a mucosal elevation was observed on the anterior wall of the middle gastric body, with a smooth surface. (b, c) Endoscopic full-thickness resection procedure: submucosal injection of normal saline+indigo carmine, incision of the edge with a dual knife, and gradual dissection with a dual knife. (d–g) Endoscopic closure procedure: there was no bleeding after the intraoperative bleeding was stopped by coagulation forceps, and the wound was sutured by purse-string suture. (h) Gastric submucosal tumour presentation.

Purse-string sutures with nylon loops and titanium clips were used to complete the endoscopic closure. Modality-specific details are given below. A titanium clip was inserted into the endoscopic clamp channel, and the first titanium clip was used to anchor the nylon rope to the edge of the wound, after which the clip was fastened as firmly as possible. Another clip was then inserted, and the previous steps were repeated until the clips were evenly distributed around the edge of the wound. The nylon ring was then tightened to ensure complete closure of the wound, and several tightened titanium clips were observed to have piled up under the endoscope. Ensuring the correct number of titanium clips needed was essential, as too many would cause the nylon rope to become too tight; this could give rise to a gap in the wound surface of the suture that could affect the healing of the wound (Figure 1).

#### 2.4.4. Laparoscopic Repair

A 1 cm arc incision was made at the inferior edge of the umbilicus to reach the subcutaneous. When the pneumoperitoneum was sufficient, the pneumoperitoneum needle was removed and a cannula needle was used to make a 10 mm puncture, which was then viewed under the laparoscope. Under laparoscopic direct vision, 10 and 5 mm trocars were used to make punctures 3 cm below the xiphoid process and the midline of the clavicle and axillary front and costal margin. The appropriate equipment was then inserted.

**Figure 2:**
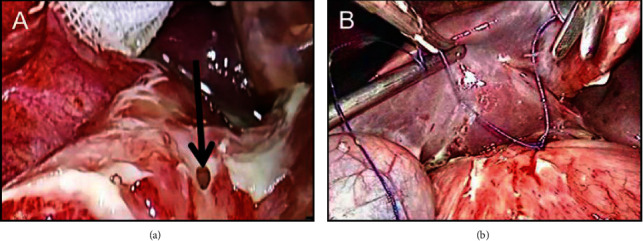
Laparoscopic repair. (a) Laparoscopic findings: gastric perforation of the patient. (b) A full-thickness suture was performed using a 3.0 absorbable suture.

The gastric content and exudates were absorbed with an attractor. After the perforation was located, a full-thickness suture was performed using a 3.0 absorbable suture (without the need for ligation), and the free part of the greater omentum was inserted into the perforation and knotted (Figure 2). The abdominal and pelvic cavities were then rinsed. A drainage tube was placed near the perforation and drawn out of the body from the anterior axillary line sleeve before being fixed. Finally, the CO_2_ was released, the casing was removed, and the skin incision was glued.

### 2.5. Postoperative Follow-Up

A postoperative examination was required one, three, and six months after surgery. The one-month follow-up primarily checked for functional recovery, including the availability of food and the presence of any obstruction, bleeding, or stenosis. The follow-ups at three and six months occurred primarily to check for recurrence. If recurrence was identified, previous judgments were reconsidered, as malignancy was likely, which required further treatment. The follow-up items mainly included gastroscopy and abdominal computed tomography; the follow-up content included the identification of any tumour recurrence/metastasis or any complications. The methods of notification for the follow-up examinations included outpatient appointments, phone calls, and SMS messages.

### 2.6. Statistical Analysis

All data collection and statistical analysis were conducted using the R (v.3.5.1) software. The differences between the two groups in terms of surgery duration, medical expenses, and inpatient days were compared using a double-sample *t*-test. The differences between the two groups in terms of surgery difficulty and effects were compared using a chi-squared (*χ*^2^) test. A *P* value of <0.05 was considered statistically significant.

## 3. Results

### 3.1. General Characteristics

A total of 120 patients were included in this study with an average age of 56.12 ± 8.89 years. There were no significant differences between the two groups in terms of age, gender, and tumour location (all *P* > 0.05). The most common tumour sites were the gastric fundus and gastric body, accounting for 42.5% and 37.5% of cases, respectively. See Table 1.

### 3.2. The Comparison of Operative and Perioperative Data between the Two Groups

**Figure 3:**
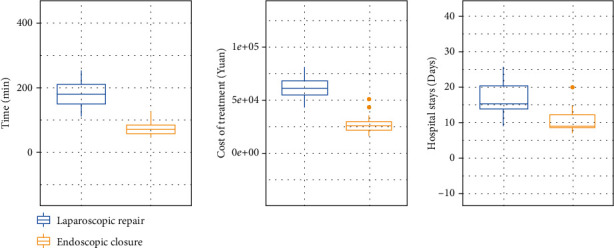
The comparison of the surgery time, cost of treatment and hospital stays between endoscopic closure and laparoscopic repair with acute perforation.

As shown in Table 2 and Figure 3, the surgery time in the endoscopic closure group was 74.7 ± 23.55 minutes, which was significantly lower compared with that in the laparoscopic repair group (178.35 ± 39.98 minutes; *P* < 0.001). In addition, the postoperative stay in the endoscopic closure group (10.5 ± 3.45) was shorter than that in the laparoscopic repair group (16.95 ± 4.58; *P* < 0.001), and the intraoperative bleeding volume and incidence of enteral nutrition initiation after surgery were significantly lower. Medical expenses were also significantly lower in the endoscopic closure group (28463.55 ± 8228.96) than in the laparoscopic repair group (61848.75 ± 8812.12; *P* < 0.001). Only one patient developed a postoperative fever in the endoscopic closure group; three patients developed a postoperative fever in the laparoscopic repair group, and one patient experienced postoperative bleeding. However, there were no statistical differences between the two groups in the incidence of R0 resection, postoperative fever, postoperative bleeding, and closure failure (all *P* > 0.05). The surgical difficulty of the two closure methods was grade four for both, according to the 2018 National Ministry of Health surgical categorisation catalogue. There were no local recurrences, distant metastases, or deaths in either of the groups during the two-year follow-up period.

## 4. Discussion

The results of this study show that when compared with laparoscopic repair, endoscopic closure reduces operative duration, intraoperative blood loss, and the risk of having enteral feeding after surgery, and it involves a shorter postoperative period. During the two-year follow-up period, neither group experienced a recurrence.

The double combined mirror is laparoscopic repair and endoscopic resection. Submucosal tumour endoscopic resection ESE/EFR in the muscularis propria requires skilled endoscopic techniques, and there is a risk that the perforation foci cannot be completely repaired [12–14]. Double combined mirror surgery for GISTs has the advantages of rapid positioning, optimisation of the surgical process, short surgery times, small wounds, small incisions, a lower risk of exogenous infection, a clear operative field, quick recovery, and a higher level of safety and effectiveness [15–17]. It is also suitable for tumours with a diameter of <5 cm, and those are difficult to locate using laparoscopic techniques. However, laparoscopic methods also have limitations; for example, laparoscopy is difficult to perform when the perforation is located in the posterior wall of the stomach and when abdominal pollution is severe and part of the operation needs to open. It also has higher requirements in terms of tumour location and cooperation among the surgical team. As such, it must only be performed by experienced physicians.

With the continued development of endoscopic resection, endoscopic perforation closure has also improved. The maturity of endoscopic resection and endoscopic closure techniques led to the development of endoscopic muscularis dissection (EMD), which includes ESE, EFR, and submucosal tunnelling endoscopic resection (STER) [18]. These procedures expand the depth and scope of an endoscopy. Endoscopic full-thickness resection allows for perforation to be successfully repaired using a microscope during surgery. For larger full-thickness defects, the omentum can be inserted into the perforated gastric cavity using strong negative pressure suction, after which metal clips can be used to clip the omentum and gastric mucosa along the defective edge and effectively suture the defect. The greatest advantage of STER technology is that it can completely remove the GIST while maintaining the integrity of the mucosal lining of the digestive tract [19]. Although perforation occurs during surgery, it can reduce the chances of a gastrointestinal fistula and intra-abdominal infection by closing the tunnel opening.

The key to the success of EMD is the site of the intraoperative perforation. Presently, with the development of endoscopic closure, the use of laparoscopic-assisted wound sutures has been gradually reduced and practice has transitioned to endoscopic closure [20], for which the technology is constantly evolving. Physicians can also receive training in ESD using acute perforation closure via animal experiments [21–23]. Furthermore, endoscopic closure reduces the number of staff and instruments required and also reduces cost and patient trauma [24–26].

In the clinical application of acute digestive tract perforation, however, endoscopic closure still faces obstacles. Accordingly, before this technology can be fully applied in clinical practice, animal experimental studies involving intraoperative acute perforation endoscopic closure and perforation repair should be undertaken by physicians. The learning curve of endoscopist technology from being unfamiliar to skilled requires standardised technical operation training, evaluation of safety and feasibility, accumulation of experience, and continuous innovation, so as to improve the overall diagnosis and treatment effect of endoscopy.

The predictability of perforation infers that it includes complications and therapeutic perforations. With the improvement of operating instruments and procedures, a new minimally invasive approach must be established for performing endoscopic closure. Passive perforation must become active perforation, and perforation itself should become a step of endoscopic resection, while the various procedures related to the gastrointestinal tract can be completed via endoscopic closure. Since gastroenteroscopy is performed on an empty stomach, it needs to be discovered in time and closed quickly with endoscopy. The symptoms of acute perforative peritonitis are mild; as such, traditional transabdominal surgery can be avoided. Furthermore, it can reduce the psychological and economic burdens on both doctors and patients, shorten the course of the disease, reduce the cost of hospitalisation, and improve the cure rate. With the development of the endoscopic closure of iatrogenic gastrointestinal perforation, the indications of endoscopic resection will continue to expand and gain clinical value [19]. In addition, the development of endoscopic suture technology infers that many perforations that were previously treated using the laparoscope-assisted endoscopic technique (LAET) can now be treated using endoscopic closure. The application of LAET will gradually be reduced and may even be replaced by EFR and STER. However, the training and promotion of endoscopic closure will require the invention and application of a variety of simple endoscopic closure instruments.

While endoscopic closure has a number of advantages over laparoscopic repair, it is vital to be aware of the potential surgical risks associated with it while doing endoscopic closure. For instance, an excessive amount of titanium clips will impede wound healing, which needs the operator to possess sufficient knowledge and experience. Moreover, the present operation has several limitations. To begin, this was not a double-blind, placebo-controlled trial. Second, it was a single-center trial; multicenter trials will be required in the future. Third, the sample size was small; future studies will require a larger trial with a higher sample size. Finally, the clinical follow-up time was brief, necessitating future research to monitor long-term clinical outcome.

## 5. Conclusion

Non-laparoscopic-assisted surgery has the advantages of reduced trauma, shorter surgery times, reduced intraoperative bleeding, and a faster recovery of gastrointestinal function. Its efficacy and postoperative recurrence rates are similar to those of laparoscopic procedures, and as such, it is worthy of clinical promotion.

## Figures and Tables

**Table 1:** Comparison of general data between the two groups.

Group	Age (years old)	Gender (male/female)	Tumour site		
			Gastric fundus	Gastric body	Gastric antrum
Endoscopic closure group (*n* = 60)	56.65 ± 7.34	34/26	29	22	9
Laparoscopic repair group (*n* = 60)	56.6 ± 10.30	39/21	22	23	15
*P* value	0.522	0.261	0.406		

**Table 2:** The comparison of endoscopic closure and laparoscopic repair with acute perforation.

	Operation methods		*P*
	Laparoscopic repair	Endoscopy closure	
Operation time span (min)	178.35 ± 39.98	74.7 ± 23.55	<0.001
Operation difficulty	Level 4	Level 4	—
Intraoperative bleeding volume (ml)	41.75 ± 34	5.6 ± 3.1	<0.001
The initiation of enteral nutrition after surgery (days)	3.5 ± 2.4	1.5 ± 0.6	<0.001
Medical expenses (yuan)	61848.75 ± 8812.12	28463.55 ± 8228.96	<0.001
R0 resection (*n*, %)	(60, 100%)	(60, 100%)	>0.05
Conversion to other procedure (*n*)	0	0	>0.05
Postoperative fever (*n*, %)	(3, 5%)	(1, 1.6%)	>0.05
Postoperative bleeding (*n*, %)	(1, 1.6%)	(0, 0.0%)	>0.05
Closure failure (*n*, %)	(0, 0.0%)	(0, 0.0%)	>0.05

## Data Availability

The datasets used and analysed during the current study are available from the corresponding author on reasonable request.
